# Tocopheryl Succinate-Induced Structural Changes in DPPC Liposomes: DSC and ANS Fluorescence Studies

**DOI:** 10.3390/molecules25122780

**Published:** 2020-06-16

**Authors:** Grażyna Neunert, Jolanta Tomaszewska-Gras, Stanislaw Witkowski, Krzysztof Polewski

**Affiliations:** 1 Department of Physics and Biophysics, Faculty of Food Science and Nutrition, Poznan University of Life Sciences, Wojska Polskiego 38/42, 60-637 Poznań, Poland; grazyna.neunert@up.poznan.pl; 2 Department of Food Safety and Quality Management, Faculty of Food Science and Nutrition, Poznan University of Life Sciences, Wojska Polskiego 31/33, 60-624 Poznań, Poland; jolanta.tomaszewska-gras@up.poznan.pl; 3 Faculty of Chemistry, University of Bialystok, Ciolkowskiego 1K, 15-245 Bialystok, Poland; wit@uwb.edu.pl

**Keywords:** DPPC liposome, phase transition, membrane permeation, differential scanning calorimetry, ANS fluorescence, tocopheryl succinate

## Abstract

Recent studies show that alpha-tocopheryl succinate (TS) exhibits selective toxicity against cancer cells. In this study, we investigated the effect of TS’s presence on the physico-chemical and structural properties of DPPC liposomes using fluorescence parameters (intensity, lifetime, and position of emission maximum) of 1-anilino-8-naphtalene sulphonate (ANS), differential scanning calorimetry (DSC) and zeta potential methods. Increasing the TS presence in the DPPC gel phase produced ANS fluorescence enhancement with a hypsochromic shift of the maximum. The zeta potential measurements show an increase in the negative surface charge and confirmed that this process is connected with the hydrophobic properties of dye, which becomes located deeper into the interphase region with a progressing membrane disorder. Temperature dependence studies showed that an increase in temperature increases the ANS fluorescence and shifts the ANS maximum emission from 464 to 475 nm indicating a shift from hydrophobic to a more aqueous environment. In the liquid crystalline phase, the quenching of ANS fluorescence occurs due to the increased accessibility of water to the ANS located in the glycerol region. The DSC results revealed that increasing the presence of TS led to the formation of multicomponent DSC traces, indicating the formation of intermediate structures during melting. The present results confirmed that TS embedded into the DPPC membrane led to its disruption due to destabilisation of its structure, which confirmed the measured biophysical parameters of the membrane.

## 1. Introduction

α-Tocopherol (Toc) is one of the most potent natural antioxidants for protecting membranes from lipid peroxidation. Additionally, Toc is known to play some important roles in the biochemical processes in cells [1,2]. When used as a drug, Tocopherol has some limitations, such as poor stability, poor bioavailability, and low accumulation in fat [3,4,5]. To overcome those deficiencies, there have been attempts to synthesise tocopherol derivatives by modifying the phenolic hydroxyl group at the chromanol ring. Esterification at this position with moieties such as oxalate, malonate, and succinate led to the formation of derivatives exhibiting strong cytotoxic potential against tumour cells [6]. Kogure [7], moreover, demonstrated that these properties are related to the presence of carboxylic groups on these moieties.

α-Tocopheryl succinate (TS) is the succinic acid ester of tocopherol and is among the most effective tocopherol derivatives that causes the apoptosis of cancer cells [8]. TS has been found to suppress tumour growth in preclinical animal studies, including those focused on breast, bladder, prostate and neck carcinomas [7,9,10,11]. TS also enhances the antitumour action of doxorubicin [12]. TS is considered as an alternative to other antitumour agents. Studies seeking to develop nanoparticle carriers loaded with doxorubicin and TS have been performed in a monolayer model [13] and in liposomes [14]. The proapoptotic properties of TS and the different forms of its liposomal delivery were reviewed by Koudelka [15].

Liposomes are widely used as a model of biological membranes to test membrane fluidity, stability and permeability in the presence of different additives. The amphiphilic nature of liposomes permits their use as drug delivery systems [16]. Phase transition behaviour is important for this property because the permeability of the bilayers to entrapped drugs increases as the bilayer becomes more fluid. Temperature is one of the main parameters determining liposomal membrane permeability. For this reason, analyses of the phase transitions induced by variations in temperature are often used for lipid bilayer characterisation. Upon heating, phospholipids, depending on water content and the type of lipid, undergo structural transformations from gel to liquid-crystalline forms. At a temperature below the transition temperature, the bilayer is rigid and impermeable, but above this temperature, it is flexible and permeable. A number of methods have been used to study these transitions, including ^13^C-NMR, EPR [17] and FTIR [18], which have provided insight into the formed structure and its rigidity. Based on the fluorescence and anisotropy methods, Mason suggested that the TS in DPPC changes its thermotropic parameters due to the presence of a four-carbon chain moiety that increases the overall length of the molecule and shifts the chromanol ring deeper into the bilayer [19,20].

The differential scanning calorimetry (DSC) method is used to determine the formation of different phase transitions and characterise the thermotropic parameters of liposomes. In calibrated DSC systems, the area under the trace is proportional to the enthalpy of transition (ΔH_m_) and includes the onset temperature of the transition (T_0_), the temperature at the peak maximum (T_m_) and the width of the transition at the half-peak height (ΔT_1/2_) [21]. DSC studies on TS in DPPC membranes have demonstrated that the presence of TS broadens the phase transition peak and lowers the temperature and onset of the phase transition. Further, Lai suggested that these observed changes are related to increased interactions between the succinate moiety and phospholipids [22].

The zeta potential (ZP) measure of the surface charge may originate from different mechanisms. The sign and magnitude of the zeta potential is determined by the net charge on the liposome’s surface. In DPPC, a zwitterion liposome, the zeta potential depends on the orientation of the polar heads of the phospholipids. At a low ionic strength, a negatively charged phosphate group determines it a small zeta potential, whereas with a high ionic strength choline group, the situation is reversed [23].

The spectroscopic properties of 1-anilino-8-naphtalene sulphonate (ANS) are complex because its quantum yield, position of emission maximum (λ_max_), fluorescence intensity (FI), and fluorescence lifetime (FL) depend on the polarity and viscosity of its microenvironment [24]. In nonpolar solvents, the FI and FL are higher than those observed in more polar solvents. The interactions of the phosphate head group atoms of lipids with charged sulfonate groups of ANS induce changes in the photophysical parameters, which allows us to explore the interactions of tocopherol derivatives embedded within phospholipids. ANS is an anionic fluorescent probe that has been used extensively in studies on the hydrophobicity of proteins [11,25]. ANS has been applied to many studies on the structural changes in liposomes and membranes induced by a variety of ligands [26,27,28,29,30,31]. ANS steady-state and time-resolved emission spectroscopy have been applied to characterise the metastable rippled gel phase [32]. The fluorescence of ANS is highly sensitive to its microenvironment; thus, conformational changes occur in the polar head group region during its interactions with ligands. The changes in ANS intensity in biological membranes were interpreted as structural changes related to transport across the membrane or as a measure of changes in transmembrane potential.

In this study, we investigated the structural and physical parameter changes of DPPC membranes induced by embedded TS, an α-tocopherol derivative characterised by a succinate tail moiety at the 6 position of the chromanol ring. TS has attracted special attention because it shows selective toxicity towards malignant cells, leaving normal cells intact [33]. To further characterise the underlying molecular mechanisms involved in the cytotoxic action of this tocopherol derivative on tumour cells [7], the ANS fluorescence and DSC methods were used. In our study we analysed three ANS fluorescence parameters FI, λ_max_, and FL, which revealed more information about the microenvironment, thus allowing determination of its hydrophobicity and the presence of water molecules in the bilayer interior. By analysing the obtained results, we were able to better understand the mechanism responsible for the interactions between the TS and DPPC liposomes leading to its disruption.

In this model study, we apply fluorescence, zeta potential, and the DSC method to explore the fluidity, ordering, structural, and thermodynamic parameters changes of DPPC liposomes under an increasing amount of embedded TS.

## 2. Results

### 2.1. Temperature Studies of ANS Fluorescence in DPPC Liposomes with Embedded TS

**Figure 1:**
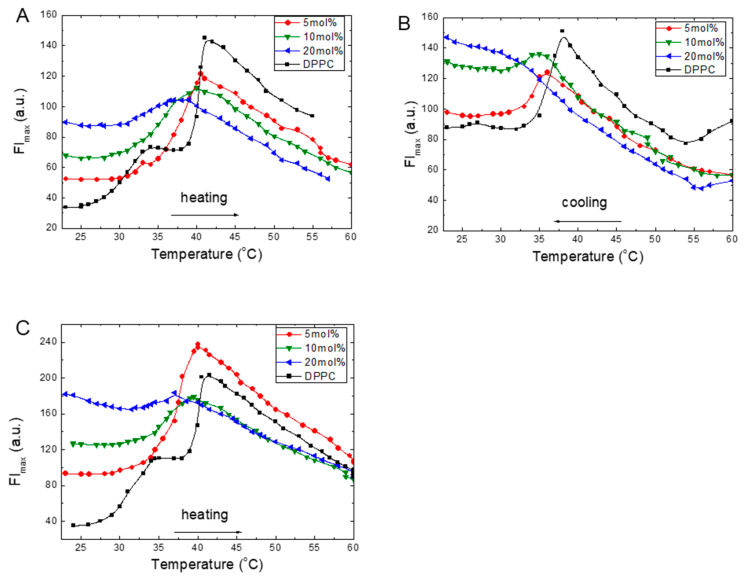
Fluorescence intensity in the maxima of emission (FI_max_) changes of 1-anilino-8-naphtalene sulphonate (ANS) (16 μM) added to the 1,2-Dipalmitoyl-sn-glycero-3-phosphocholine (DPPC) (0.08 mg/mL) with an increasing concentration of α-Tocopheryl succinate (TS) during heating (**A**), cooling (**B**) or α-Tocopherol (Toc) during heating (**C**).

The temperature dependencies of the fluorescence intensity at the emission maximum (FI_max_) of the ANS in the DPPC liposomes with increasing amounts of TS or Toc are provided in Figure 1. In pure DPPC, during heating, the FI_max_ of ANS changes significantly (Figure 1A–C). In the gel phase at 20 °C, the FI_max_ was low, and it increased with an increase in temperature. This growth was not linear but accelerated to a local maximum at 35 °C and reached the maximum value at 42 °C. Further heating of the sample to 60 °C caused the FI_max_ to monotonically decrease. The obtained curve demonstrated two maxima at 35 and 42 °C. These temperatures are very close to the characteristic values obtained from the calorimetric method, from which the DSC measurements were labelled as the pretransition temperature (T_0_) and the main phase transition temperature (T_m_) [34].

Temperature scans of the ANS FI_max_ from the DPPC liposomes with increasing embedded concentrations of TS during heating are given in Figure 1A. At 5 mol% of TS in DPPC, the gel phase FI_max_ is greater than that measured for DPPC. Starting at 32 °C, a continuous, sharp FI_max_ increase is observed up to a maximum at 40.5 °C, and the subsequent intensity exhibits a monotonic decrease. The trace obtained for 10 mol% of TS is much wider; its intensity is reduced, and its maximum is shifted to 39 °C. However, in the gel phase at 25 °C, the FI_max_ is higher than the 5 mol% TS concentration. In the presence of 20 mol% of TS in the DPPC, a significant increase of FI_max_ in the gel phase is observed. However, the trace was flattened, its intensity was reduced, and the ANS FI_max_ maximum value was shifted to 37 °C. Further temperature increase up to 60 °C caused a monotonic decrease in the FI_max_ intensity.

We also carried out similar measurements during the cooling of the samples from 60 to 20 °C. The plots of these measurements for TS are given in Figure 1B. The results reveal a good recovery of the traces during the heating of the samples, including recovery of their shapes, whereas the positions of the FI_max_ maxima were shifted to lower temperatures. The observed hysteresis during the heating–cooling cycles indicates the structural changes formed during cooling of the sample. Similar thermal hysteresis was reported for DPPC multilamellar dispersions using deuterium magnetic resonance [35]. Such a lack of thermal convergence between the main phases was ascribed to the formation of the metastable ripple or ripple-gel mixed phases [36]. This process was observed for pure DPPC liposomes and in simulation studies on reverse melting during heating and cooling, which indicated the formation of melting seeds during this process [37]. Moreover, in the gel phase after cooling, all FI_max_ values were 2- to 3-fold higher than the intensities observed at the beginning of the heating process (Figure 1A). These findings suggest that, during cooling, a part of the ANS molecules were trapped inside the bilayer structure. An increase in the ANS’s intensity in the ripple gel phase during cooling has also been reported, and this phenomenon was ascribed to the formation of a metastable rippled gel phase [32].

As a reference for the results obtained with TS, we examined Toc (Figure 1C). At 5 mol% of Toc in the DPPC in the gel phase, the FI_max_ was twice that measured for pure DPPC. Starting at 30 °C, a continuous FI_max_ increased was observed to a maximum located at 40 °C. A subsequent monotonic decrease was then observed. The traces obtained under higher Toc concentrations of 10 and 20 mol% revealed that, in the gel phase, there were significant increases in the FI_max_ intensities, while the FI_max_ maxima were shifted to 39 and 37 °C, respectively. This shape was flattened compared to the shapes recorded at lower Toc concentrations. After reaching the maxima, monotonic decreases in FI_max_ were observed up to 60 °C were observed.

Some common features were observed on the traces of TS and Toc during the heating of the samples (Figure 1A,C). At 5 mol% concentrations, the temperature dependencies were similar, including the absence of the shoulder present on the pure DPPC trace at 35 °C, the shift of the main phase transition peak to a lower temperature, and an increase in FI_max_ in the gel phase. After reaching the maximum, all traces exhibited a monotonic decrease in FI_max_ with an increase in temperature up to 60 °C. In all measured samples, the greatest FI_max_ of ANS was observed at T_m_. Increases in temperature above T_m_ led to monotonic FI_max_ intensity decreases with comparable slopes for all samples, which suggests that a similar mechanism was responsible for the observed phenomenon.

### 2.2. Kinetics of ANS Adsorption on DPPC in the Presence of Tocopherol Derivative

**Figure 2:**
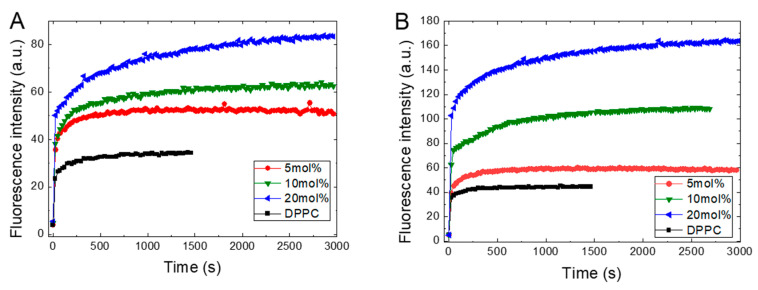
Kinetics of ANS’s (16 μM) inclusion into the DPPC (0.8 mg/mL) membrane at 25 °C in the presence of TS (**A**) or Toc (**B**). TS and Toc concentrations are given in the legend.

Figure 2 presents the kinetics of ANS fluorescence intensity (FI) during its binding to the DPPC liposomes in the gel phase at 25 °C with increasing concentrations of TS or Toc in its structure. Figure 2 shows that the binding of the ANS molecules to pure DPPC membranes occurs almost instantly, as observed by the rapid increase in FI, which subsequently remains at a constant level. Under increasing TS or Toc concentrations, we observe a fast, proportionally increasing FI and then the appearance of slowly growing components that finally reach plateau.

The kinetics of ANS inclusion shows the biphasic character, since added ANS always instantaneously increases its FI followed by slower growth, leading at longer times, to equilibration in the system. We fit the observed kinetics to two models available from Origin: a double exponential component model and a bidose response model. In both models, we obtained two components using calculations, one very fast and another much slower. The obtained times for the fast component are around a tenth of a second. We assume that the fast component originates from the electrostatic interactions called ion pairing between the sulfonate group of the ANS and the polar spot on the bilayer surface, leading to adsorption of the ANS molecules from bulk water onto the membrane surface. For pure DPPC, this mechanism is supported by the low fluorescence intensity and red shifted emission maximum of ANS. Additionally, since this fast component is present in all samples and does not depend on added ligands, it indicates that a diffusion-controlled mechanism is involved in this process. The increasing intensity observed in the presence of TS or Toc reflects the increasing number of formed binding sites. The longer time of the second component, which is in the range of hundreds of seconds, suggests that this second component arises from the ANS molecules translocating deeper into the inner leaflets of the bilayer due to an interruption of the membrane structure caused by the presence of embedded TS. As the concentration of TS increases, this TS leads to an increasing number of binding places, which are ultimately observed as an increase in fluorescence intensity. Similar conclusions, as well as the assignment of both components of the biphasic characteristics of ANS’s binding to liposomes based on time-resolved emission studies, were previously reported, where a lifetime of 50–100 ms was ascribed to the short component, while the long-time component represents the transport of dye molecules into the inner layer of the bilayer [32,38,39].

Figure 2 shows that recorded ANS fluorescence kinetics differ between TS and Toc in their absolute magnitude and shape. These differences arise from the different numbers of ANS molecules binding to the possible accessible places on/in the liposomes and are related to induced structural changes in the DPPC membrane due to embedded tocopherol or its derivative. However, the main factor that governs the accessibility for the binding places is the electrostatic potentials of the membrane’s surface [23]. The TS, which possesses an anionic succinate moiety, is present in the bilayer, and together with the negatively charged phosphate group in the phospholipid head group form an electrostatic obstacle that repels the negatively charged sulfonate group in the ANS, which slows the penetration of the ANS into the bilayer. Thus, Toc, with its short hydroxyl moiety, causes much less interference in the ANS’s penetration into the DPPC and binds to more places formed during the conformational changes induced by Toc.

### 2.3. Temperature Dependencies of the ANS Emission Maxima (λ_max_)

**Figure 3:**
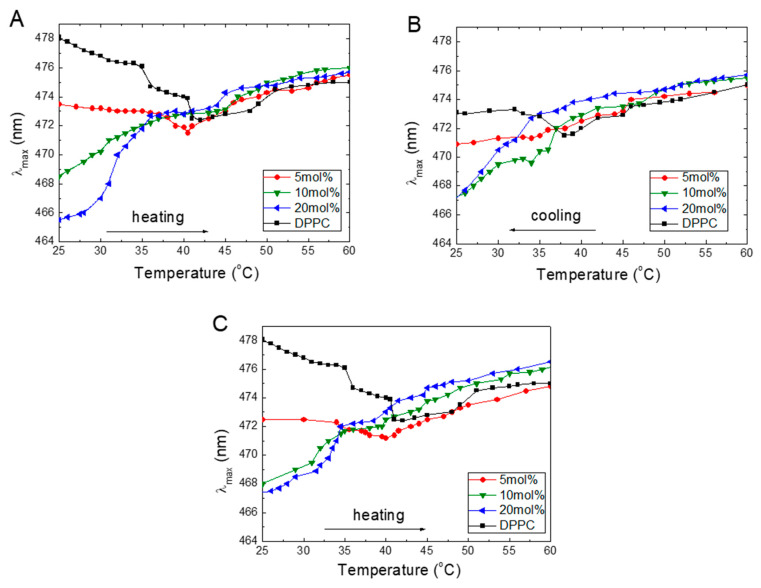
Temperature dependences of the positions of the ANS (16 μM) fluorescence maxima (λ_max_) in DPPC (0.08 mg/mL) for TS during heating (**A**), cooling (**B**), and for Toc during heating (**C**).

The emission maxima of the ANS bound to the liposome are blue-shifted compared to free dye in the solution. Since the position of the emission maximum (λ_max_) is related to the ANS’s local microenvironment, its location may be used to probe the changes in the DPPC that occur during its interactions with TS or Toc. During the temperature experiments, the emission spectra of the ANS revealed that, besides the varying intensity, the position of the maximum also shifted. The relations between the temperature (during heating and cooling) and λ_max_ of the ANS at different concentrations of TS and Toc are plotted in Figure 3.

Figure 3 shows that, for pure DPPC in the gel phase at 25 °C, the λ_max_ of ANS occurs at 478 nm, which locates the molecule in the membrane interphase layer. An increasing temperature shifts the λ_max_ to a shorter wavelength indicating that the ANS molecules move to a more hydrophobic environment [40]. Starting at 30 °C, λ_max_ decreases steeply with a small plateau between 35 and 40 °C; finally, λ_max_ reaches its lowest value of 471 nm, which is observed at 41 °C. Further temperature increase shifted λ_max_ in a near monotonic manner back to 475 nm as observed at 60 °C. These findings indicate that, in the DPPC liquid phase, the ANS molecules experience a more aqueous environment.

Figure 3A shows that increasing the concentrations of TS embedded into the DPPC in the gel phase at 25 °C shift the λ_max_ from 478 to 466 nm at 20 mol% TS, which means that the ANS molecules moved from the interphase to a less aqueous environment. Increasing the temperature causes a shift of the λ_max_ to longer wavelengths for all TS concentrations. At 5 mol% TS, the maximum in still observed at a temperature of 40.5 °C, strongly suggesting, the main phase transition. At 10 and 20 mol%, the steep increase in the wavelength with an increase in temperature is observed, and no phase transition is detected. A further temperature increase above 40 °C resulted in slow monotonic increases, finally reaching the λ_max_ at 475 nm under a temperature of 60 °C. In the liquid phase, the λ_max_ values for all concentrations were very similar (around 475 +/– 1 nm).

The presence of Toc in DPPC (Figure 3C) revealed the influence of an increase in the temperature on λ_max_, similar to that observed for TS. Increasing concentrations of Toc in DPPC in the gel phase also led to a decrease in λ_max_, which indicates that an increasing number of ANS molecules transfer from an aqueous environment into the membrane structure. Further heating above T_m_ monotonically shifted the λ_max_ to longer wavelengths, which were characteristic of a more aqueous environment. The only difference we noted was the much wider distribution of λ_max_ at 60 °C compared to TS.

Presumably, during heating, the ANS molecules populate accessible binding sites in a manner that reaches a maximum at the temperature of the main phase transition. The observed shifts of the λ_max_ to longer wavelengths with an increase in temperature indicate that a more aqueous environment may arise from the two mechanisms. First, this change might be connected to the withdrawal of ANS from occupied places inside the membrane as the temperature increases. Second, an increased penetration of water at higher temperatures into the membrane structure due to the loosening of the membrane may occur. To further examine this problem, we performed experiments in which the samples were cooled from 60 to 20 °C.

The plots obtained during cooling of the samples (Figure 3B) showed existing reversibility to those obtained during heating, indicating the cooperative character of the investigated processes; however, a 5-nm down-shift was observed at λ_max_ for DPPC. At lower temperatures, when the sample was in the gel phase, the λ_max_ shifted again to shorter wavelengths, which suggests that during cooling, the ANS molecules were trapped inside the hydrophobic environment in the membrane.

### 2.4. Fluorescence Lifetime of ANS in DPPC

The fluorescence lifetime (FL) of the ANS in lipids depends on the dielectric environment of the fluorophore. Even small changes in ANS hydration in its microenvironment due to the interactions with water molecules can significantly alter the FL [41]. The measured FLs of ANS in the DPPC membranes in the gel phase were in the range of 7.5–8.0 ns; with increasing TS concentrations, the FL remained constant. At 50 °C in the fluid phase, an increase in TS causes a rupture of the DPPC structure, which leads to an increasing presence of the aqueous phase, as sensed by that probe and FLs, which were in the range of 5.5–6.0 ns and did not significantly change with increasing concentrations of TS. During the cooling of the samples from 60 to 20 °C in the gel phase, the lifetime increased from 5.5 to 8.0 ns. At the same time, a few-fold higher FI_max_ values were observed compared to the FI_max_ at the gel phase in Figure 1A, which confirms the entrapment of the ANS molecules inside the membrane interior. These data demonstrate that the presence of TS caused an increased influx of water molecules that led to hydration and further fluidisation of the bilayer at lower temperatures. Presumably, this effect is connected to the increasing volume of the head groups in the interface region of the bilayer due to the presence of embedded TS in the membrane structure.

### 2.5. ANS Parameters Calculations

**Figure 4:**
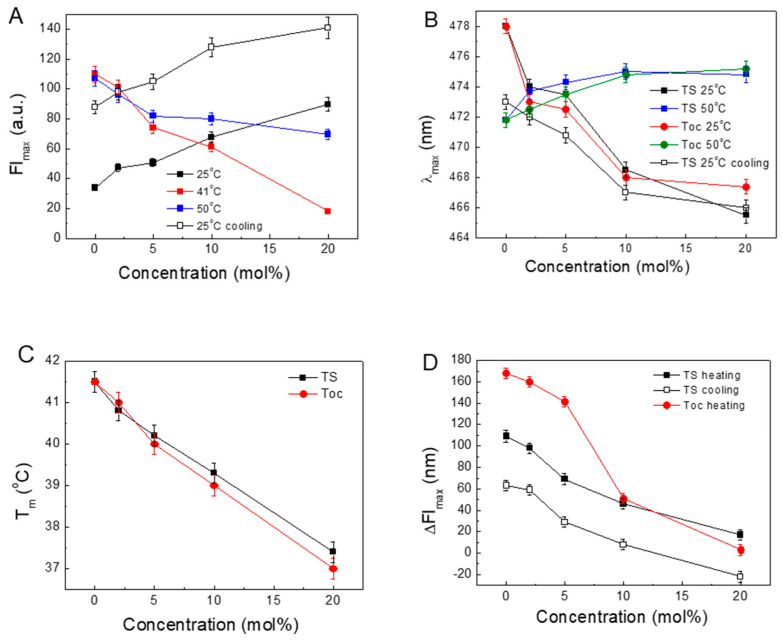
Changes in the ANS parameters observed during the heating/cooling of DPPC with embedded TS or Toc. The TS fluorescence maximum intensities (FI_max_) at 25, 41 and 50 °C (**A**). Positions of the fluorescence maxima (λ_max_) with an error of ± 0.5 nm, at 25 and 50 °C (**B**). Shifts in the temperature at the fluorescence maximum (T_m_) with increasing concentrations of TS and Toc (**C**). Decreasing amplitude of ANS intensity (ΔFI_max_) (**D**). ΔT = 0.25 °C.

The obtained results allowed us to calculate some parameters to characterise the observed process of the influence of TS on the DPPC structure. The changes in the parameters obtained from the above-presented results are shown in Figure 4. The plots present the relations of FI_max_, λ_max_, T_m_ and the amplitude of intensity (ΔFI_max_), defined as difference between FI_max_ at T_m_ and FI_max_ at 25 °C, versus the concentrations of TS or Toc.

Figure 4A shows the changing fluorescence intensities in the gel phase, liquid phase and at T_m_ with increasing concentrations of TS. In the gel phase at 25 °C, the intensity increases by nearly three-fold compared to the DPPC, whereas in the fluid phase, the FI_max_ decreases. This quenching is possibly related to the increasing influx of water into the inner volume of the liposome. Moreover, the FI_max_ recorded at 25 °C during cooling shows an intensity three to two times higher than that observed at the beginning of the heating procedure.

Figure 4B shows that by increasing the concentration of TS or Toc in the gel phase, the λ_max_ shifts to lower values, indicating that the ANS molecules reside in progressively more hydrophobic environments. In the liquid phase, λ_max_ is located around 474 nm with a negligible influence of tocopherols concentration, indicating that, in this phase, the ANS molecules reside in a more aqueous environment.

Our preliminary studies on the ANS in solvents with different dielectric constants and protic properties (data not shown) revealed that a decrease in permittivity causes a blue shift of λ_max_ with simultaneous increases in the FI and FL. This fluorescence intensity enhancement with a hypsochromic shift arises from the interaction of the ionic group of phospholipids with the sulfonate anionic group of the ANS. This interaction reduces the intermolecular charge transfer rate, which leads to an increase in FI [42,43].

Figure 4C presents the relationships between T_m_ and the concentrations of TS and Toc. For both tocopherols, a linear decrease with similar slopes was observed. Since the main phase temperature depends on the structure of the acyl chains, in the presence of this same phytol, a hydrophobic part of tocopherols induced similar results.

Figure 4D displays the changes in ΔFI_max_ with an increase in the TS or Toc concentrations. In pure DPPC, the permeation of the ANS shows the biggest ΔFI_max_ value. This is in agreement with the previously published results showing that the highest transfer of ligands through the membrane occurs at T_m_ [44,45,46]. This demonstrates that increasing the presence of both tocopherols decreases the ΔFI_max_ of ANS during its transfer to the interior of the bilayer.

The observed quenching indicates the increasing presence of water molecules in the interphase region of the bilayer. The observed shift of λ_max_ to longer wavelengths with increasing temperature, especially at higher concentrations of TS, suggests an increase of water penetration into the structurally modified membrane rather than the efficient squeezing-out of the ANS molecules from the membrane’s interior. This observation is more pronounced when we compare this observation with the results in Figure 1B, in which, during cooling, the FI_max_ in the gel phase was much higher than the initial values before heating.

### 2.6. Zeta Potential Measurements

**Figure 5:**
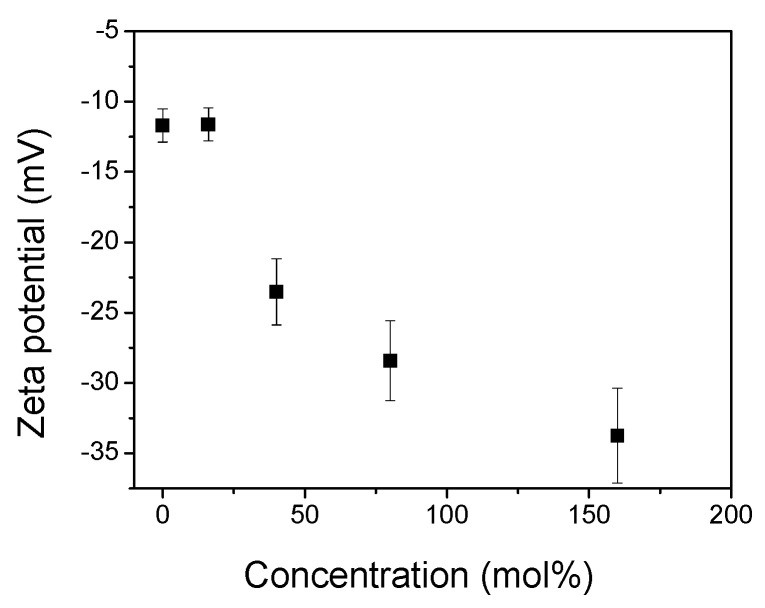
Zeta potential (ZP) of the DPPC liposomes (0.08 mg/mL) versus an increasing TS concentration. Error bars are equal ± SD calculated for three different repetitions.

In order to determine the interactions between the ANS and membrane surface in the gel phase, we measured the zeta potential (ZP) of the DPPC surface versus an increase in the TS concentration (Figure 5). The DPPC liposome has a zwitterionic nature, and its polar heads may reorient depending on ionic strength. Thus, in buffer solutions at a low ionic strength, the liposomes present a small negative potential due to the exposed phosphate group, whereas under a high ionic strength, the ZP is slightly positive [23]. The presented plot indicates that for pure DPPC, the zeta potential is −11.7 mV, which is similar to the values presented in previous researches [47,48,49,50]. By increasing the amount of embedded TS, the negative zeta potential rises. Given this behaviour, increasing the number of TS molecules with a negatively charged succinate moiety adds to the total surface potential. From Figure 1A shows that the ANS fluorescence intensity in the gel phase increases with a rising mole fraction of the TS. An increasingly negatively charged surface should repel a negatively charged ANS molecule, thereby decreasing its intensity. Thus, the increase in the ANS fluorescence intensity suggests that the bonding of ANS with the membrane surface cannot be explained in terms of electrostatic interactions. This indicates another mechanism related to the hydrophobic interaction between the ANS and DPPC bilayer.

### 2.7. DSC Measurements

The above-presented ANS fluorescence results provided knowledge about important parameters of the membranes, giving insight into the possible mechanism behind the observed structural changes. To obtain more information and confirm our assumptions regarding these structural changes, we applied DSC measurements. Using this method, we were able to obtain T_m_, the width of the transition at the half-peak height (ΔT_1/2_) and cooperativities of the phase transitions, as well as add some thermotropic information to our study on the influence of TS on the stability of DPPC membranes.

**Figure 6:**
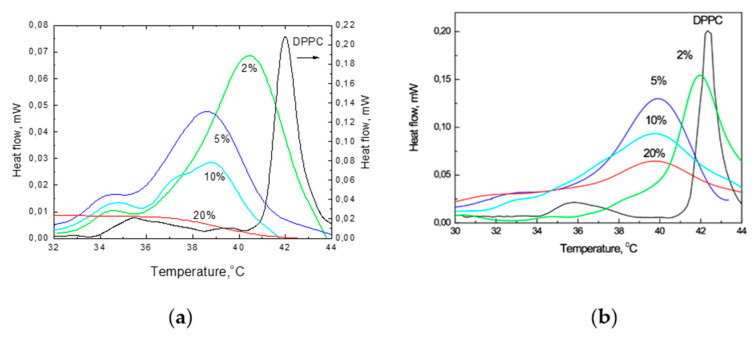
Thermograms of mixtures of DPPC with 0, 2, 5, 10, and 20 mol% of TS (the heat flow scale on the right is related to pure DPPC only) (**a**) and Toc at 0, 2, 5, 10 and 20 mol% (**b**).

The results of the DSC studies of the influence of TS on the phase transitions of DPPC are given in Figure 6. These results demonstrate that the incorporation of TS at 2, 5, 10 and 20 mol% into the DPPC membranes significantly changed the DSC traces of these mixtures. Even at a low 2 mol% concentration of TS, the observed peak becomes highly unsymmetrical towards lower temperatures. Additionally, the broadening of the main transition peak (where ΔT_1/2_ increased from 0.47 °C to 2.59 °C), decreased T_m_ from 42.35 to 40.5 °C, reduction in the intensity of the whole trace and the appearance of a shoulder at 34.5 °C were observed. The presence of 5 mol% TS led to a further reduction in the T_m_, broadening of the whole trace and a further decrease in intensity. At 10 mol%, the peak height decreased significantly (by approximately two-fold). Moreover, three shoulders at 39, 37 and 34 °C appeared, which indicates the composed character of the trace. The incorporation of 20 mol% TS led to a decrease of the main phase T_m_ from 42.1 to 37.5 °C; widening of ΔT_1/2_ from 0.61 to 3.45 °C, with a simultaneous decrease in the onset temperature; enthalpy of the phase transition (ΔH_m_) decrease from 32.6 to 6.53 kJ mol^−1^; and a decreased maximum intensity of the peak. Another thermotropic parameter known as the cooperativity unit (CU) [51], decreased from 238 (for pure DPPC) to 37 at 5 mol% of TS, indicating the increased disorder in the bilayer [52].

In pure DPPC during heating, a small peak was observed at 35 °C, which was ascribed to the so-called pretransition and related to the formation of a ripple phase. The increased presence of TS led to its disappearance from the trace. The observed broadening of ΔT_1/2_, progressively decreasing T_m_ and diminishing ΔH_m_ and cooperativity, together with the simultaneously formed new bands, indicate that the structural changes in the liposomes that were induced by the presence of the TS molecules were direct symptoms of the shift of the fluidisation process to lower temperatures in the presence of TS.

**Figure 7:**
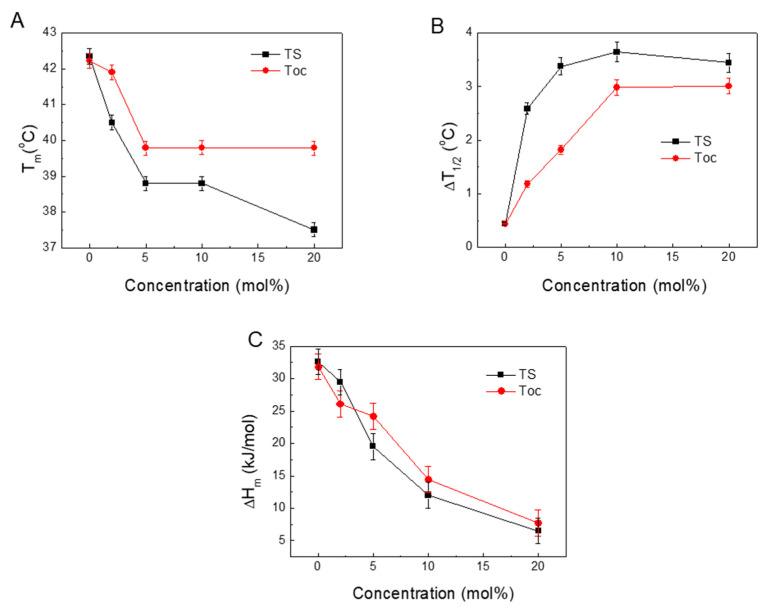
Plots of the parameters calculated from the DSC thermograms: temperatures of the main transition (T_m_) (**A**), half-width of the peak (ΔT_1/2_) (**B**), enthalpy of the main transition (ΔH_m_) (**C**).

Figure 7 presents the relations between the measured and calculated parameters derived from the DSC scans of T_m_, ΔT_1/2_ and ΔH_m_ along with the changing TS concentrations.

Figure 7A shows the relationships between the shifts of the T_m_ and the concentrations of TS incorporated into DPPC. At low concentrations, the T_m_ changes for Toc and TS are similar. Differences appear at higher concentrations of TS when the formation of a new phase occurs.

Figure 7B shows that increasing the presence of embedded TS or Toc significantly widened the ΔT_1/2_ of the main peak. Because this parameter is related to the cooperativity of the phase transition, an increase in its value indicates the progressive disappearance of cooperativity [52].

Figure 7C shows that the ΔH_m_ of the main phase transition decreased by increasing the TS concentration in the DPPC. This reduction in ΔH_m_ indicates that increasing concentrations of TS induced structural changes in the DPPC liposomes that led to a loosening of the membrane structure, thereby leading to weaker interactions between the acyl chains in the phospholipids. This also increased the mobility of the acyl chains and thus increased the disorder, which led to a decrease in the T_m_ and a widening of the ΔT_1/2_. The calorimetric parameters (T_m_, ΔT_1/2_ and ΔH_m_) that were obtained from the DSC traces are sensitive indicators of the physical phenomena that occurred in the system. The observed changes can be attributed to order and disorder in the packing of the phospholipid molecules, the cooperativity of the main transition and fluidisation in the system.

## 3. Discussion

In this study, we applied ANS fluorescence and DSC methods to identify the structural changes induced by the presence of TS in DPPC membranes. Both methods delivered information facilitated elucidation of the mechanisms of the observed changes. The ANS emission parameters are sensitive to the physical state of the bilayer, and this phenomenon was exploited to retrieve the structural changes induced by the presence of ligands. Apart from the thermodynamic parameters, the DSC data also confirmed structural changes in the DPPC induced by increasing the presence of TS.

**Figure 8:**
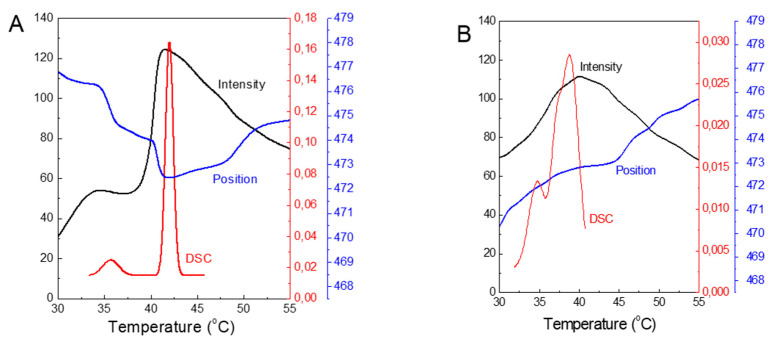
Summary plot of the ANS FI_max_ - Intensity (a.u.) and their λ_max_ - Position (nm) and the DSC trace of DPPC (μW) versus temperature observed during the heating of the samples: for pure DPPC (**A**) and for DPPC with 10 mol% of TS (**B**).

To demonstrate the feasibility of using ANS fluorescence to study structural changes in DPPC membranes, we combined the plots of the measured parameters obtained from both methods (Figure 8). For pure DPPC, both plots of ANS fluorescence parameters show local maxima at 35 and 42 °C, which mimic the temperatures characteristic of the pretransition and main phase transition obtained from the DSC. Thus, the effect of added TS on the membrane structure, as determined by applying ANS, shows very good agreement with the results obtained from the DSC studies.

The obtained temperature relations of FI_max_ and λ_max_ of the ANS gave more information beyond that obtained previously from the steady-state or lifetimes measurements [26,32]. The plots of λ_max_ indicate that, in the gel phase, the binding of the ANS molecules to the membrane was accompanied by their shift from the polar region of the interphase with a dielectric constant ε of 30–40 to a more hydrophobic environment within an ε range of 25–30. At the DPPC fluid phase, the plot revealed decreasing FI_max_ and hydrophobicity, suggesting that the ANS molecules experience a more aqueous environment. The steady-state and time-resolved ANS fluorescence results confirmed that the observed quenching was due to an increasing influx of water molecules into the bilayer.

The sharp increase in ANS FI_max_ in the temperature regions before the pretransition and main phase transition indicates on structural change in the membrane. Similar results have been reported for the permeation of other dyes and drugs [38,43]. The results reported by Tsong on ANS binding to the phospholipid bilayer structures indicated that the process occurs via two kinetic unimolecular phases, one phase in a range of 50 ms (weak but observable within all measured ranges) and a slower phase in a range of seconds. The fast component is related to the binding and reorientation of the probe on the surface, while the slow one represents the transport of the dye into the inner layer of the lipid vesicle. This second process is very sensitive to temperature and exhibits its maximum at the temperature of the main phase transition [38]. In another study Tsong showed that the decreasing intensity of ANS in bilayer structures is modulated by the presence of cholesterol in a concentration-dependent manner [42]. Similarly, Jacobsen and Papahadjopoulos observed that the inner monolayer is not accessible to the ANS at temperatures below T_m_ because the membrane is in a gel state and increasing the temperature allows the dye to bind to the inner monolayer [43].

The observed correlation between the increased ANS fluorescence with increasing TS content in the gel phase suggests a mechanism of electrostatic interactions between the dye and the phospholipid head group atoms. However, the results from the zeta potential studies indicate that the driving forces increasing ANS intensity are hydrophobic interactions. Due to the structural changes in DPPC induced by the presence of TS, the additional sites for ANS inclusion are exposed. It is known that modifications in the interface region may also induce alterations of the membrane structure. Membrane alterations have also been reported in alcohol-induced phospholipids [53,54,55]. These interactions increase the volume of the head group in the interface, which leads to a rearrangement of acyl chain packing due to an increase in the hydrocarbon tilt and molecular area. For phospholipids with the same saturated acyl chains, increasing the head group volume influences the packing geometry of the acyl chains. In the gel phase position of ANS emission maximum is at 475 nm, indicating that the ANS is located in a more aqueous region due to the presence of bulk water molecules. Weak fluorescence arises from a small number of binding places on the membrane’s surface. This suggests that the binding of the ANS to the membrane in the gel phase occurs via the adsorption of the ANS molecule on the membrane surface at the lipid/water interface. This process occurs very fast. An increase in temperature leads to the first phase changes known as pretransition and the formation of the ripple phase. During pretransition, an ordered flat membrane transforms into a periodically undulating bilayer. This process is connected to bilayer reorganisation, which allows more ANS molecules to move into the interface layer, thus increasing the FI intensity, with the FL in the range of 8 ns. Both parameters indicate that more ANS molecules moved into a more hydrophobic microenvironment. Further temperature increase leads to a main phase transition where the membrane is transformed from rippled ordered state to a fluid phase, which makes the bilayer less ordered. This phase is characterised by the different packing of its acyl chains and the increase in the surface area of the lipid head group, offering the possibility to increase the influx of water molecules into the bilayer sensed by the ANS molecules and decrease its FL. Additionally, the disorder introduced by the presence of TS in the membrane in the liquid-crystalline phase afforded an increase in the amount of water molecules that flow into the interphase region, thus significantly changing the hydrophobic–hydrophilic balance inside the membrane, which is crucial for membrane stability. Thus, to fully understand the apoptogenic activity of TS, the presence of water should be added to previously determined factors, such as presence of a chromanol ring with a chargeable moiety at the C6 position, as the factors that destabilise phospholipid membranes [6,7,20].

DSC data confirmed the formation of additional mixed phase structures and the lowering of T_m_ in the presence of TS. The shift in the T_m_ occurs due to intermolecular dynamics connected with the presence of ligand molecules in the membrane. Acyl chain melting starts from a seed present in the system, which, in our case, was a single TS molecule. The present results show that increasing the presence of TS or Toc in DPPC strongly affects its phase properties, similar to the observed temperature induced phase transitions.

**Figure 9:**
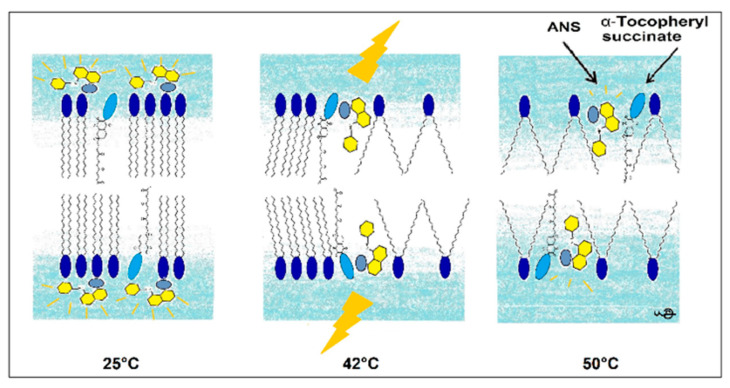
Pictorial presentation of structural changes in the DPPC membrane induced by TS reported by ANS fluorescence.

In this research, we used the fluorescence parameters of FI_max_, λ_max_, FL, and the kinetics of binding to acquire a more complete picture compared to the previously applied ANS FI or its FL. This complex approach allowed us to determine the mechanism of changes in the DPPC membrane induced by the presence of TS. A pictorial presentation of the proposed mechanism is presented in Figure 9.

## 4. Materials and Methods

### 4.1. Chemicals

**Figure 10:**
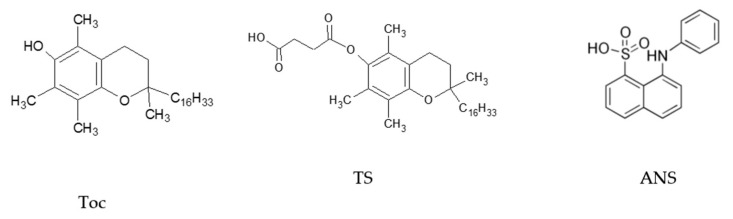
Chemical structures of α-tocopherol (Toc), its ester derivative succinate (TS) and 1-anilino-8-naphtalene sulphonate (ANS).

1,2-Dipalmitoyl-sn-glycero-3-phosphocholine (DPPC), 1-anilino-8-naphtalene sulphonate (ANS) and chloroform (spectroscopic grade) were purchased from SIGMA Chemical Co. (SIGMA Chemical Corp., St.Louis, MO, USA). DL-α-tocopherol (Toc) and DL-α-Tocopheryl succinate (TS) were newly synthesised according to previously reported procedures [56,57]. Double deionised water produced with a MicroPure Water System (TKA, Niederelbert, Germany) was used as a solvent. The chemical structures of the tocopherols and ANS are shown in Figure 10.

### 4.2. Preparation of Vesicles

For liposome preparation, dry DPPC and the studied tocopherol ester or Toc were dissolved in chloroform and mixed in the required proportions (the final concentrations of tocopherols were 0–20 mol% or 0–160 mol% for the zeta potential measurements). Next, the solvent was removed under a vacuum at 50 °C for 15 min with an R-125 rotary evaporator (Büchi Labortechnik AG, Flawil, Switzerland). The formed DPPC dry film was hydrated with double-distilled deionised water (5 mL in volume, pH 5.3, conductivity < 60 nS/cm) and vortexed for 30 min at 50 °C. For the DSC measurements the resulting liposomal suspension (2 mg/mL final phospholipid concentration) was dispersed for 2 min by ultrasonication in an ultrasonic bath and stored at 0–4 °C for at least 12 h before the measurements. For the ANS fluorescence measurements, the final concentration of lipids was 0.08 mg/mL. Next, all samples were extruded repeatedly eleven times through a 100 nm pore polycarbonate filter using a LiposoFast Basic LF-1 extruder (Avestin, Mannheim, Germany). The particle size distribution in the liposome suspension and the polydispersity index (Pdl) were determined using a method of dynamic light scattering (DLS) with a Zetasizer Nano (Malvern Instruments, Worcestershire, UK) at 20 °C under an angle of 90°. The zeta potential (ZP) was measured using the same Zetasizer of Malvern Instruments at 20 °C. The ZP was calculated from the electrophoretic mobility using the Helmholtz–Smoluchowski equation. The processing was run by the software included within the system.

The mean values of the liposome sizes, 160, 120, and 105 nm, were determined via an analysis of the intensity, volume and number of peaks, respectively, and did not change significantly in the presence of TS. Moreover, the low values observed for Pdl, ranging between 0.10 and 0.20, revealed a good degree of homogeneity among the investigated systems.

During the measurements, the lipid/ANS ratio was around 100:1 or less, which is substantially lower than the binding capacity of DPPC for ANS. The obtained results demonstrate that the bound ANS introduced negligible changes to the measured physico-chemical and thermotropic properties of DPPC. Similar conclusions were previously published [32].

### 4.3. Spectroscopic Measurements

The steady-state emission spectra were obtained using a Shimadzu RF 5001PC fluorimeter (Shimadzu Corp., Kyoto, Japan) with an excitation wavelength of 380 nm. All spectroscopic measurements were performed in a 1 × 1 cm quartz cuvette at a temperature range of 20 to 60 °C. The temperature of the sample in the fluorimeter was controlled using a temperature unit adapter. During the heating or cooling of the samples in the above-mentioned range, the emission spectra of ANS were measured every 2 °C.

### 4.4. Differential Scanning Calorimetry (DSC)

Differential scanning calorimetry (DSC) was performed with a DSC 7 (Perkin Elmer Corp., Norwalk, CT, USA) equipped with an Intracooler II and the Pyris Software 10.1. Details on these experiments were published previously [51]. The sample pan was placed in the calorimeter and isothermally held at 10 °C for 5 min and subsequently heated to 60 °C with a scanning rate of 2 °C min^−1^. Three replicates were analysed for each sample. The parameters of the peak temperature (T_m_), enthalpy (ΔH_m_, J g^−1^) and cooperativity unit (CU) were determined from the DSC curve.

### 4.5. Fluorescence Lifetimes

The ANS fluorescence lifetime measurements were carried out with a TimeHarp 200 PC-board (PicoQuant, Berlin, Germany) for time-correlated single photon counting with a resolution of 27 ps per channel. The excitation source was a coaxial sub-nanosecond flashlamp 5000 F (IBH, Glasgow, England) filled with nitrogen, with maximum emissions cantered at 337 nm and a 1.3 ns wide pulse with full width at half maximum (FWHM). A 337 nm XL30 interference filter from Laser Components (Laser Components, Olching, Germany) was used as the excitation window to avoid leaking from the nitrogen spectrum. The emissions were measured with a PMA 182 photon sensor head (PicoQuant, Berlin, Germany). The data were analysed by an exponential reconvolution method using a nonlinear least-squares fitting program. The quality of the fit was characterised in terms of the residual distribution and reduced χ^2^ values.

### 4.6. Fitting Procedures

All plots, figures and calculation procedures, including statistics of plotted data, were prepared using the Origin program (OriginLab Corp., MA, USA, ver. 8.5). All experiments were repeated at least in triplicate.

## 5. Conclusions

The presented ANS fluorescence and DSC results confirmed that the incorporation of TS into the DPPC membrane led to changes in its structural and thermotropic properties. Increasing the presence of TS in the membrane gel phase lowered the acyl chain packing order, thereby loosening the structure, which allowed the ANS molecules to more deeply penetrate into the interphase of the membrane. In the liquid-crystalline phase, increasing the TS concentration decreases the cooperativity and formation of multicomponent structures thus allowing the influx of water molecules into the disrupted DPPC structure leading to progressive fluidisation of the membrane at lower temperatures. Thus, the formation of mixed structures between components leading to interrupter transport through the membrane and increasing the penetration of water into the inner bilayer structure may be possible mechanisms responsible for the effective disruption of cell membranes caused by the presence of TS.
